# New Benzil and Isoflavone Derivatives with Cytotoxic and NO Production Inhibitory Activities from *Placolobium vietnamense*

**DOI:** 10.3390/molecules27144624

**Published:** 2022-07-20

**Authors:** Lien T. M. Do, Tuyet T. N. Huynh, Jirapast Sichaem

**Affiliations:** 1 Institute of Environment-Energy Technology, Sai Gon University, Ho Chi Minh City 748355, Vietnam; liendo.ieet@sgu.edu.vn (L.T.M.D.); lamhuynh1579@gmail.com (T.T.N.H.); 2 Lu Gia Secondary School, Distr. 11, Ho Chi Minh City 70000, Vietnam; 3 Research Unit in Natural Products Chemistry and Bioactivities, Faculty of Science and Technology, Thammasat University Lampang Campus, Lampang 52190, Thailand

**Keywords:** *Placolobium vietnamense*, placovinones A–D, benzil and isoflavone derivatives, cytotoxicity, NO production inhibition

## Abstract

The phytochemical investigation of *Placolobium vietnamense* stems led to the isolation of a new isoflavone derivative (**1**) and three new benzil derivatives (**2**–**4**), together with four known pyranoisoflavones (**5**–**8**). The structures of all isolated compounds were determined on the basis of extensive spectroscopic analyses, including NMR and HRMS spectral data, as well as comparison of their spectroscopic data with those reported in the literature. The cytotoxicity of all isolated compounds was assessed against the human liver hepatocellular carcinoma (Hep G2) cell line, and compound **1** displayed the most significant cytotoxicity with an IC_50_ value of 8.0 μM. Furthermore, all isolated compounds were also tested for their inhibitory activity against NO production in RAW 264.7 macrophages. Of these, compound **1** exhibited the strongest inhibitory efficacy against the LPS-induced NO production with the IC_50_ value of 13.7 μM.

## 1. Introduction

*Placolobium* is a genus of plants in the family Fabaceae, which contains three accepted species. These are distributed throughout the world’s tropical regions, some extending into temperate zones, especially in East Asia [1]. *Placolobium vietnamense* N.D.Khoi & Yakovlev is an indigenous plant species, known in Vietnam as ‘Rang Rang’. It is a perennial tree with a straight, cylindrical trunk, and brown bark. The fruit is a small pod with a single seed. This plant is used as a folk remedy for snakebites, debility, and to increase strength after childbirth [1]. There has only been one investigation into the chemical constituents of *P. vietnamense* [1]. Previously, our group reported the isolation and structure elucidation of six isoflavonoids, including afrormosin, cladrastin, 8-*O*-methylretusin, millesianin C, barbigerone, and durallone from the EtOAc stem extract of this plant, together with their cytotoxicity. Encouraged by structurally diverse bioactive compounds from *Placolobium* species [2], the aim of this investigation is to revisit *P. vietnamense* in order to search for new bioactive compounds. We report herein the isolation and characterization of benzil and isoflavone derivatives from the stems of *P. vietnamense*. All isolated compounds were assessed for their cytotoxicity against human liver hepatocellular carcinoma (Hep G2) cell line, which is one of the most fatal cancers and has spread to the liver from other organs. Additionally, the inhibitory activity toward NO production in RAW 264.7 macrophages of all isolated compounds was also evaluated.

## 2. Results and Discussion

### 2.1. Structural Elucidation of the Isolated Compounds

**Figure 1:**
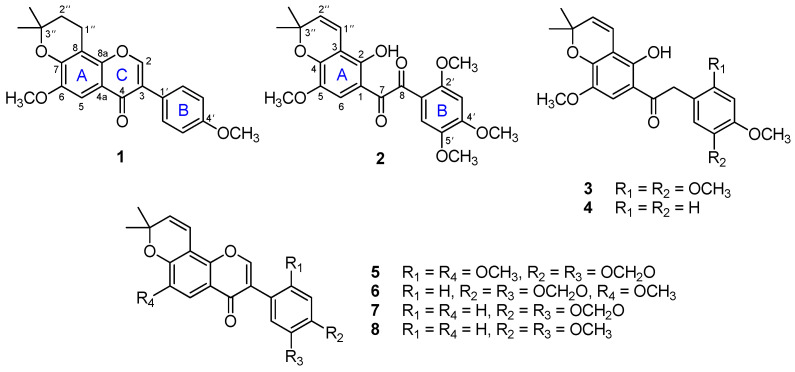
Chemical structures of **1**–**8**.

Chromatographic separation of benzil and isoflavone derivatives from *P. vietnamense* stems allowed for the isolation of eight compounds, including a new isoflavone derivative, placovinone A (**1**), and three new benzil derivatives, placovinones B-D (**2**–**4**), along with four known pyranoisoflavones (**5**–**8**) (Figure 1). The structures of all isolated compounds were elucidated based on NMR and HRMS spectral data, as well as a comprehensive comparison of their spectroscopic and physical data with values from the published literature. The known isolated pyranoisoflavones were characterized as ichthynone (**5**) [3], durmillone (**6**) [4], calopogoniumisoflavone B (**7**) [5], and 4′,5′-dimethoxy-6,6-dimethylpyranoisoflavone (**8**) [6].

**Figure 2:**
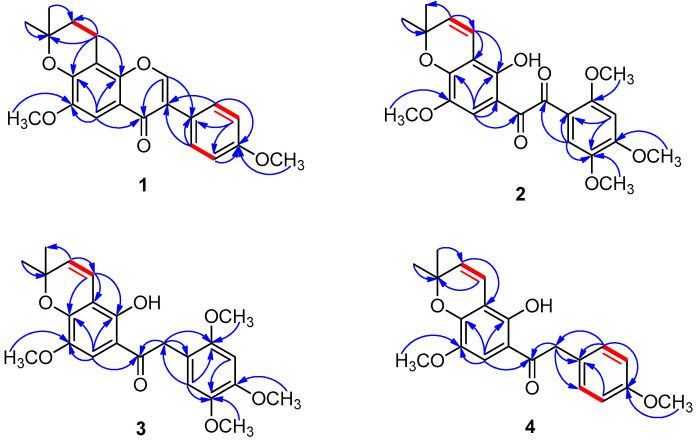
Key COSY (red bold line) and HMBC (blue arrow) correlations of **1**–**4**.

Compound **1** was isolated as a colorless gum. The HRESIMS revealed a protonated molecular ion peak at *m/z* 367.1549 [M + H]^+^ (calcd for C_22_H_23_O_5_ 367.1545) corresponding to the formula C_22_H_22_O_5_. The ^1^H NMR signal at *δ*_H_ 8.42 (s, H-2) and ^13^C NMR signal at *δ*_C_ 152.7 (C-2) were characteristic of the isoflavone skeleton [7]. The existence of AA′BB′ spin-system indicated *para*-substituted B-ring. The presence of a 2,2-dimethyldihydropyrano [8] and two methoxy substituents was identified from the ^1^H and ^13^C NMR spectral data (Table 1). A singlet resonance at *δ*_H_ 7.32 was assigned to the aromatic proton H-5 on the basis of the long-range coupling to C-4 (*δ*_C_ 174.4), C-7 (*δ*_C_ 148.3), and C-8a (*δ*_C_ 109.9), observed in the HMBC spectrum (Figure 2). The methoxy group at *δ*_H_ 3.84 (s) was assigned as 6-OCH_3_ according to the HMBC correlation between 6-OCH_3_ and C-6 (*δ*_C_ 147.1). The long-range correlations observed in the HMBC spectrum of H-1″ (*δ*_H_ 2.87, t, *J* = 6.5 Hz) to C-7 and C-8a were key correlations that revealed the position of 2,2-dimethyldihydropyrano moiety was fused to C-7 and C-8, with the anticipated oxygenation at C-7 being supported by the HMBC correlation from H-5 to C-7. Its 1D and 2D NMR spectral data were similar to those of 6-methoxycalopogonlum isoflavone A [9], except for the replacement of a double bond at C-1″ and C-2″ of the 2,2-dimethylpyrano substituent in 6-methoxycalopogonlum isoflavone A by a C-C single bond in **1**. Based on the above spectral evidence, the structure of **1** was established and trivially named as placovinone A.

Compound **2** was obtained as a white amorphous powder. Its molecular formula was determined to be C_23_H_24_O_8_ based on a protonated molecular ion peak at *m/z* 429.1566 (calcd for C_23_H_25_O_8_ 429.1549). The signal of a hydroxyl group at *δ*_H_ 10.11 (s, 2-OH) in the ^1^H NMR spectrum, together with those of two carbonyl groups at *δ*_C_ 190.7 (C-7) and 191.4 (C-8) in the ^13^C NMR spectrum, indicated that **2** was a derivative of 1,2-diphenyl-1,2-ethanedione [10]. The ^1^H and ^13^C NMR spectral data (Table 2) further revealed the presence of a 2,2-dimethylpyrano fragment and four methoxy substituents. In the ^1^H NMR spectrum, two singlet protons at *δ*_H_ 6.76 and 7.41, were assigned to the two *para*-positioned aromatic protons H-3’ and H-6’ of the B-ring [11], indicating the B-ring of **2** with 2′,4′,5′-trimethoxy substituent. This was also supported by the strong correlations in the HMBC spectrum (Figure 2). The singlet of the aromatic proton at *δ*_H_ 7.24 was identified as H-6 on the basis of the HMBC correlations from H-6 to C-1 (*δ*_C_ 112.5), C-2 (*δ*_C_ 149.7), C-4 (*δ*_C_ 148.2), and C-7 (*δ*_C_ 190.7). Consequently, the remaining methoxy group (*δ*_H_ 3.84, s) was located at C-5, confirmed by the key HMBC correlation between 5-OCH_3_ and C-5 (*δ*_C_ 142.5). Hence the location of the 2,2-dimethylpyrano moiety was found to be at C-3 (*δ*_C_ 108.9) and C-4, with the anticipated oxygenation at C-4 being confirmed by the HMBC correlation from H-6 to C-4. A careful comparison of the ^1^H and ^13^C NMR spectral data (Table 2) of **2** with dielsianone [12] identified similar signals, distinguished by the presence of two methoxy groups at C-2′ and C-5′. The existence of these two methoxy substituents was confirmed by the HMBC correlations from 2′-OCH_3_ (*δ*_H_ 3.33, s) and 5′-OCH_3_ (*δ*_H_ 3.89, s) to C-2′ (*δ*_C_ 156.8) and C-5′ (*δ*_C_ 155.6), respectively (Figure 2). From the aforementioned results, the structure of **2** was identified and named as placovinone B.

Compound **3** was isolated as a white amorphous powder. Its molecular formula, C_23_H_26_O_7_, was determined from its protonated molecular ion peak at *m/z* 415.1759 [M + H]^+^ (calcd for C_23_H_27_O_7_ 415.1757). This was further confirmed by the ^1^H and ^13^C NMR spectral data, which disclosed one methylene, two methyl, two olefinic, three aromatic methine, four methoxy, and ten quaternary carbons (Table 2). The spectroscopic ^1^H and ^13^C NMR patterns of **3** were very similar to those of **2**, with the only difference being that the keto carbonyl group at C-8 in **2** (*δ*_C_ 191.4) was replaced by a methylene substituent in **3**. This deduction was supported by the HMBC correlations from H-8 (δ_H_ 4.20, s) to C-7 (*δ*_C_ 202.9) and C-1′ (*δ*_C_ 114.2). Based on the above spectral evidence, compound **3** was identified and named placovinone C.

Compound **4** was obtained as a white amorphous powder. The molecular formula C_21_H_22_O_5_ was obtained from its HRESIMS, which showed a protonated molecular ion peak at *m/z* 355.1553 [M + H]^+^ (calcd for C_21_H_23_O_5_ 355.1545). ^13^C NMR and HSQC spectra of **4** indicated 21 signals, including one carbonyl, one methylene, two methyl, two methoxy, seven methine, and eight quaternary carbons. Two signals at *δ*_H_ 7.21 (d, *J* = 8.7 Hz, H-2′, 6′) and 6.88 (d, *J* = 8.7 Hz, H-3′, 5′) appearing as an AA′BB′ type confirmed the presence of a simple *para*-substituted B-ring, with a methoxy group (*δ*_H_ 3.47, s) being positioned at C-4′ (*δ*_C_ 158.2). The careful comparison of the ^1^H and ^13^C NMR spectral data (Table 2) of **4** was shown to be similar to those of **3**, differing only in the absence of two methoxy groups at C-2′ (*δ*_C_ 130.7) and C-5′ (*δ*_C_ 114.1) on the B-ring of **4**, which was supported by the COSY and HMBC correlations (Figure 2). On the basis of these spectral data, the structure of **4** was unambiguously established and named as placovinone D.

### 2.2. Cytotoxicity

The cytotoxicity of each isolated compound against Hep G2 cell line was assessed [13,14,15] and the IC_50_ values are listed in Table 3. Compounds **1**–**8** exhibited different degrees of cytotoxicity toward Hep G2 cell line. Among them, compound **1** exhibited the most significant cytotoxicity against Hep G2 cell line against Hep G2 cell line with an IC_50_ value of 8.0 μM. Compounds **2**–**4** and **8** showed moderate cytotoxicity with the IC_50_ values of 19.8, 22.9, 23.4, and 35.6 μM, respectively, while compounds **5**–**7** exhibited weak cytotoxicity with the IC_50_ values of 99.1, 71.6, and 66.6 μM, respectively. Based on the above cytotoxic results, the presence of the 2,2-dimethyldihydropyrano ring in the case of **1** might be responsible for enhancing the activity.

### 2.3. Inhibition of Nitric Oxide Production

To determine the inhibitory effects of the isolated compounds on NO production (Table 3), LPS-stimulated RAW 264.7 cells were treated with various concentrations of tested compounds [16]. Additionally, the viability of RAW 264.7 cells using an MTT assay to avoid the cytotoxic effects of the isolated compounds was evaluated. Among eight isolated compounds, compounds **1** and **4** highly inhibited NO production in RAW 264.7 cells with the IC_50_ values of 13.7 and 15.5 μM, respectively, whereas compounds **2**, **3**, and **8** moderately inhibited NO production with the IC_50_ values of 31.0, 47.4, and 54.7 μM, respectively. Compounds **1** and **4** demonstrated cytotoxicity toward RAW 264.7 cells with the IC_50_ values of 79.2 and 42.6 μM, respectively, while most of the other compounds showed no obvious cytotoxicity (IC_50_ >100 μM). These results demonstrate that the presence of the *para*-substituted B-ring of **1** and **4** might be responsible for inhibiting NO production.

## 3. Materials and Methods

### 3.1. General Experimental Procedures

The NMR spectra were recorded on Bruker AvanceNEO 600 MHz and Bruker Avance III™ HD 500 MHz NMR spectrometers in DMSO-*d*_6_ (Merck, Darmstadt, Germany). Optical rotations were measured on a A.KRÜSS Optronic P8000 polarimeter (KRÜSS, Hamburg, Germany). The IR data were obtained with a Jasco 6600 FT-IR spectrometer using an ATR technique (Jasco, Japan). The HRESIMS spectral data were generated with a X500_R_ QTOF model mass spectrometer (Sciex, Framingham, MA, USA) and Dionex Ultimate 3000 HPLC system hyphenated with a QExactive Hybrid Quadrupole Orbitrap MS (Thermo Fisher Scientific, Waltham, MA, USA). Silica gel 70–230 mesh (Merck) and Sephadex LH-20 gel (GE Healthcare Bio-Sciences AB, Uppsala, Sweden) were used for column chromatography. 

### 3.2. Plant Material

The stems of *P. vietnamense* were collected in Dak Nong province, Vietnam, in February 2017. The plant material was identified by botanist Vo Van Chi (former lecturer at the University of Medicine and Pharmacy, Ho Chi Minh City, Vietnam). A voucher specimen (No. SGU-A001) has been deposited in the Herbarium of the Laboratory of Chemistry-Biology-Environment, Sai Gon University, Ho Chi Minh City, Vietnam.

### 3.3. Extraction and Isolation

The air-dried *P. vietnamense* stems (23 kg) were powdered prior to being extracted with 95% EtOH (45 L × 5) at room temperature. The filtered solution was concentrated in vacuo to afford EtOH crude extract (1200 g). This crude extract was suspended in water and partitioned with *n*-hexane and then EtOAc to yield *n*-hexane (271.2 g) and EtOAc (301.3 g) extracts, respectively. The *n*-hexane extract was subjected to silica gel column chromatography (CC) and eluted with *n*-hexane–EtOAc (9:1–0:10, *v/v*) and then EtOAc–MeOH (10:0–0:10, *v/v*). Based on their TLC behavior, the eluted fractions were grouped into fractions HEX.1–HEX.7. Fraction HEX.4 (34.5 g) was subjected to further silica gel CC and eluted with *n*-hexane–EtOAc (8:2, *v/v*) to give subfractions HEX.4.1–HEX.4.8. Subfraction HEX.4.1 (3.0 g) was subjected to silica gel CC and eluted with *n*-hexane–EtOAc (85:15, *v/v*) to yield **3** (7.0 mg), **5** (8.0 mg), and **6** (9.7 mg). Subfraction HEX.4.2 (0.9 g) was further purified using silica gel CC and eluted with *n*-hexane–EtOAc (8:2, *v/v*) to yield **2** (6.5 mg), **7** (6.4 mg), and **8** (11.4 mg). Subfraction HEX.4.3 (1.1 g) was selected for further purification using Sephadex LH-20 gel CC and eluted with MeOH to afford **1** (5.8 mg) and **4** (6.4 mg).

Placovinone A (**1**). Colorless gum. UV (CH_3_OH) *λ*_max_ (log *ε*) 210 (4.49), 231 (4.25), 278 (4.81), 334 (3.47) nm; IR (ATR) *ν*_max_ 2975, 1718, 1619, 1457, 1343, 1279, 1203, 1150, 1013, 757 cm^−^^1^; HRESIMS *m/z* 367.1549 [M + H]^+^ (calcd for C_22_H_23_O_5_ 367.1545); ^1^H NMR (DMSO-*d*_6_, 500 MHz) and ^13^C NMR (DMSO-*d*_6_, 125 MHz) see Table 1.

Placovinone B (**2**). White amorphous powder. UV (CH_3_OH) *λ*_max_ (log *ε*) 250 (4.39), 270 (4.72), 296 (4.30), 337 (3.18) nm; IR (ATR) *ν*_max_ 3392, 2977, 2904, 1713, 1635, 1451, 1372, 1288, 1246, 900 cm^-1^; HRESIMS *m/z* 429.1566 [M + H]^+^ (calcd for C_23_H_25_O_8_ 429.1549); ^1^H NMR (DMSO-*d*_6_, 600 MHz) and ^13^C NMR (DMSO-*d*_6_, 125 MHz) see Table 2.

Placovinone C (**3**). White amorphous powder. UV (CH_3_OH) λ_max_ (log ε) 205 (4.07), 272 (4.87), 339 (2.98) nm; IR (ATR) ν_max_ 3394, 2977, 2889, 1710, 1642, 1447, 1333, 1289, 1216, 763 cm^−1^; HRESIMS *m/z* 415.1759 [M + H]^+^ (calcd for C_23_H_27_O_7_ 415.1757); ^1^H NMR (DMSO-*d*_6_, 600 MHz) and ^13^C NMR (DMSO-*d*_6_, 125 MHz) see Table 2.

Placovinone D (**4**). White amorphous powder. UV (CH_3_OH) λ_max_ (log *ε*) 205 (4.11), 270 (4.87), 333 (3.06) nm; IR (ATR) *ν*_max_ 3395, 2977, 2896, 1712, 1643, 1448, 1339, 1287, 1218, 763 cm^−1^; HRESIMS *m/z* 355.1553 [M + H]^+^ (calcd for C_21_H_23_O_5_ 355.1545); ^1^H NMR (DMSO-*d*_6_, 600 MHz) and ^13^C NMR (DMSO-*d*_6_, 125 MHz) see Table 2.

### 3.4. Cytotoxicity Assay

According to a previous procedure [17], the cytotoxic evaluation of **1**–**8** against the growth of human hepatocellular carcinoma (Hep G2) cell line was carried out. The positive control was ellipticine, a powerful anticancer medication with various modes of action. The cancer cells were grown in Dulbecco’s Modified Essential Medium (DMEM) at 37 °C in a 5 % CO_2_ environment with 10% fetal bovine serum (FBS), 1% penicillin and streptomycin, and 1% L-glutamine. The investigated compounds were added at concentrations ranging from 0.5 to 128 µg/mL by dissolving in DMSO (20 mg/mL), and the incubation was carried out once more for 72 h under the same conditions. Following the procedure, an MTT solution (10 µL, 5 mg/mL) was added to each well. The percentage of cell viability vs. sample concentration was plotted using SigmaPlot 10 (Systat Software Inc., San Jose, CA, USA) to calculate the IC_50_ values.

### 3.5. Inhibition of Nitric Oxide Production Assay

#### 3.5.1. Cell Culture

RAW 264.7 cells were stocked in Dulbecco’s Modified Essential and grown at the condition of 37 °C in DMEM supplemented with 10% heat-inactivated FBS, streptomycin sulfate (100 µg/mL), and penicillin (100 units/mL) in a humidified environment of 5% CO_2_. The RAW 264.7 cells were pre-incubated every two days.

#### 3.5.2. Cell Viability Assay on RAW 264.7 Cells

The cell viability assay was used to determine the cytotoxic effect of the isolated compounds on RAW 264.7 cells. At a density of 1 × 10^5^ cells per well, RAW 264.7 cells were seeded on a 96-well plate and allowed to adhere for 4 h. Then, the cells were treated with 0.5% DMSO, celastrol, and isolated compounds at the indicated concentrations. Celastrol was used as a positive control [16]. After incubating 24 h, the viable cells were measured with a colorimetric assay based on the mitochondria’s ability in viable cells to reduce MTT [18]. The viability cells were treated with vehicle only and were defined as 100% viable. [OD_570_ (treated cell culture) × 100]/OD_570_ was the formula used to determine the percentage of macrophage surviving cells after treatment (vehicle control).

#### 3.5.3. Measurement of Nitric Oxide (NO) Production

The RAW 264.7 cells were stimulated with or without 1 μg/mL of LPS (lipopolysaccharide), which was purchased from Sigma Chemical Co. (St. Louis, MO, USA), for 24 h with or without 0.5% DMSO, celastrol, and isolated compounds at the indicated concentrations. The culture supernatant (100 μL) was then reacted with 100 μL of Griess reagent [16]. After the Griess assay, the remaining cells were used to screen for their viability using colorimetric assay-MTT (Sigma Chemical Co., St. Louis, MO, USA).

## 4. Conclusions

In conclusion, we have conducted the successful isolation of eight compounds, including a new isoflavone derivative (**1**) and three new benzil derivatives (**2**–**4**), together with four known pyranoisoflavones (**5**–**8**) from *P. vietnamense* stems. To the best of our knowledge, compounds **1**–**8** were isolated for the first time from the genus *Placolobium*. The biological evaluations showed that **1** exhibited the most significant cytotoxicity toward Hep G2 cell line and the strongest inhibitory activity against the LPS-induced NO production. According to these investigation results, the structure of **1** is a promising candidate and could be used as a template for discovering potential anticancer and anti-inflammatory agents.

## Figures and Tables

**Table 1:** ^1^H (500 MHz) and ^13^C (125 MHz) NMR spectroscopic data of **1** recorded in DMSO-*d*_6_ (*δ* in ppm).

Position	*δ*_H_ (*J* in Hz)	*δ*_C_	Position	*δ*_H_ (*J* in Hz)	*δ*_C_
2	8.42, s	152.7	3′	6.99, d (8.3)	113.6
3		122.8	4′		158.9
4		174.4	5′	6.99, d (8.3)	113.6
4a		115.9	6′	7.52, d (8.3)	130.0
5	7.32, s	101.8	1″	2.87, t (6.5)	16.4
6		147.1	2″	1.87, t (6.5)	30.6
7		148.3	3″		75.9
8		109.9	4″	1.35, s	26.3
8a		149.5	5″	1.35, s	26.3
1′		124.4	6-OCH_3_	3.84, s	55.1
2′	7.52, d (8.3)	130.0	4′-OCH_3_	3.79, s	55.5

**Table 2:** ^1^H (600 MHz) and ^13^C (125 MHz) NMR spectroscopic data of **2**–**4** recorded in DMSO-*d*_6_ (*δ* in ppm).

Position	2		3		4	
	*δ*_H_ (*J* in Hz)	*δ*_C_	*δ*_H_ (*J* in Hz)	*δ*_C_	*δ*_H_ (*J* in Hz)	*δ*_C_
1		112.5		110.8		110.8
2		149.7		153.6		154.1
3		108.9		109.3		109.5
4		148.2		149.1		149.5
5		142.5		140.8		141.1
6	7.24, s	108.7	7.43, s	113.0	7.41, s	113.7
7		190.7		202.9		203.3
8		191.4	4.20, s	38.8	4.26, s	43.6
1′		114.8		114.2		126.9
2′		156.8		151.3	7.21, d (8.7)	130.7
3′	6.76, s	98.0	6.70, s	98.4	6.88, d (8.7)	114.1
4′		143.6		142.5		158.2
5′		155.6		140.8	6.88, d (8.7)	114.1
6′	7.41, s	110.0	6.83, s	115.7	7.21, d (8.7)	130.7
1″	6.55, d (9.9)	116.0	6.58, d (9.9)	115.1	6.56, d (9.6)	113.7
2″	5.63, d (9.9)	130.0	5.75, d (9.9)	129.1	6.74, d (9.6)	129.3
3″		76.8		77.8		78.0
4″	0.92, s	26.3	1.14, s	27.4	1.39, s	27.9
5″	0.92, s	26.3	1.14, s	27.4	1.39, s	27.9
5-OCH_3_	3.84, s	56.1	3.76, s	56.1	3.76, s	56.5
2′-OCH_3_	3.33, s	56.7	3.73, s	56.2		
4′-OCH_3_	3.55, s	55.9	3.67, s	56.3	3.47, s	55.2
5′-OCH_3_	3.89, s	56.1	3.79, s	55.8		
2-OH	10.11, s		12.76, s		12.76, s	

**Table 3:** Cytotoxicity against Hep G2 cells and inhibition of NO production in macrophage RAW 264.7 cells of **1**–**8**.

Compound	Cytotoxicity (IC_50_, µM) ^a^	NO Production (IC_50_, µM) ^a^
**1**	8.0 ± 0.2	13.7 ± 0.5
**2**	19.8 ± 1.5	31.0 ± 0.3
**3**	22.9 ± 0.5	47.4 ± 0.3
**4**	23.4 ± 0.5	15.5 ± 0.4
**5**	99.1 ± 0.9	>100
**6**	71.6 ± 0.6	>100
**7**	66.6 ± 0.5	>100
**8**	35.6 ± 0.3	54.7 ± 0.2
Ellipticine ^b^	0.43 ± 0.03	
Celastrol ^b^		1.00 ± 0.10

^a^ IC_50_ values were expressed as the mean values of three experiments ± SD. ^b^ Positive control.

## Data Availability

The supporting information can be found in the Supplementary Materials.

## Supplementary Materials

The following supporting information can be downloaded at: https://www.mdpi.com/article/10.3390/molecules27144624/s1, Figures S1–S23: HRESIMS, 1D, and 2D NMR spectra of **1**–**4**.

## Sample Availability

Samples of the compounds are not available from the authors.
