# Supplementary material for: New Benzil and Isoflavone Derivatives with Cytotoxic and NO Production Inhibitory Activities from Placolobium vietnamense
Source: Molecules. 2022 Jul 20;27(14):4624. doi: 10.3390/molecules27144624 (PMC9317696; doi:10.3390/molecules27144624)
Supplement: Supplementary file 1 [file molecules-27-04624-s001.zip › molecules-1782444-supplementary.pdf]

Supplementary Materials

New Benzil and Isoflavone Derivatives with Cytotoxic and NO Production Inhibitory Activities from *Placolobium vietnamense*

Lien T. M. Do [1], Tuyet T. N. Huynh [1,2], Jirapast Sichaem [3*]

[1] Sai Gon University, Ho Chi Minh City, 748355, Vietnam; liendo.ieet@sgu.edu.vn
[2] Lu Gia secondary school, Distr. 11, Ho Chi Minh City 70000, Vietnam; lamhuynh 1579@gmail.com
[3] Research Unit in Natural Products Chemistry and Bioactivities, Faculty of Science and Technology, Thammasat University Lampang Campus, Lampang 52190, Thailand; jirapast@tu.ac.th

* Correspondence: jirapast@tu.ac.th; Tel.: +66-54237986

Contents	Page
**Figure S1.** The ^{1}H NMR spectrum of **1** in DMSO-*d*$_6$	2
**Figure S2.** The ^{13}C NMR spectrum of **1** in DMSO-*d*$_6$	2
**Figure S3.** The COSY spectrum of **1** in DMSO-*d*$_6$	3
**Figure S4.** The HSQC spectrum of **1** in DMSO-*d*$_6$	4
**Figure S5.** The HMBC spectrum of **1** in DMSO-*d*$_6$	5
**Figure S6.** The HRESIMS spectrum of **1**	6
**Figure S7.** The ^{1}H NMR spectrum of **2** in DMSO-*d*$_6$	7
**Figure S8.** The ^{13}C NMR spectrum of **2** in DMSO-*d*$_6$	7
**Figure S9.** The HSQC spectrum of **2** in DMSO-*d*$_6$	8
**Figure S10.** The HMBC spectrum of **2** in DMSO-*d*$_6$	9
**Figure S11.** The HRESIMS spectrum of **2**	10
**Figure S12.** The ^{1}H NMR spectrum of **3** in DMSO-*d*$_6$	11
**Figure S13.** The ^{13}C NMR spectrum of **3** in DMSO-*d*$_6$	11
**Figure S14.** The COSY spectrum of **3** in DMSO-*d*$_6$	12
**Figure S15.** The HSQC spectrum of **3** in DMSO-*d*$_6$	13
**Figure S16.** The HMBC spectrum of **3** in DMSO-*d*$_6$	14
**Figure S17.** The HRESIMS spectrum of **3**	15
**Figure S18.** The ^{1}H NMR spectrum of **4** in DMSO-*d*$_6$	16
**Figure S19.** The ^{13}C NMR spectrum of **4** in DMSO-*d*$_6$	16
**Figure S20.** The COSY spectrum of **4** in DMSO-*d*$_6$	17
**Figure S21.** The HSQC spectrum of **4** in DMSO-*d*$_6$	18
**Figure S22.** The HMBC spectrum of **4** in DMSO-*d*$_6$	19
**Figure S23.** The HRESIMS spectrum of **4**	20

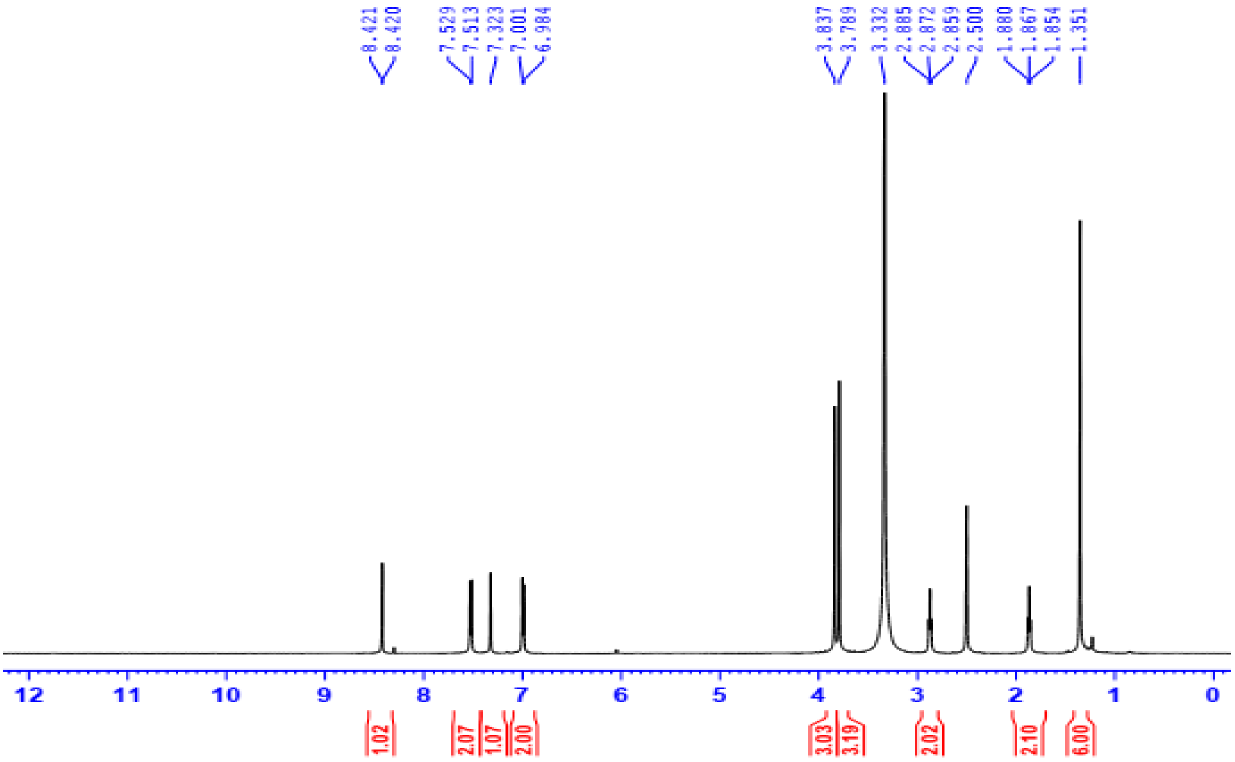

Figure S1. The ^{1}H NMR spectrum of **1** in DMSO-d_6

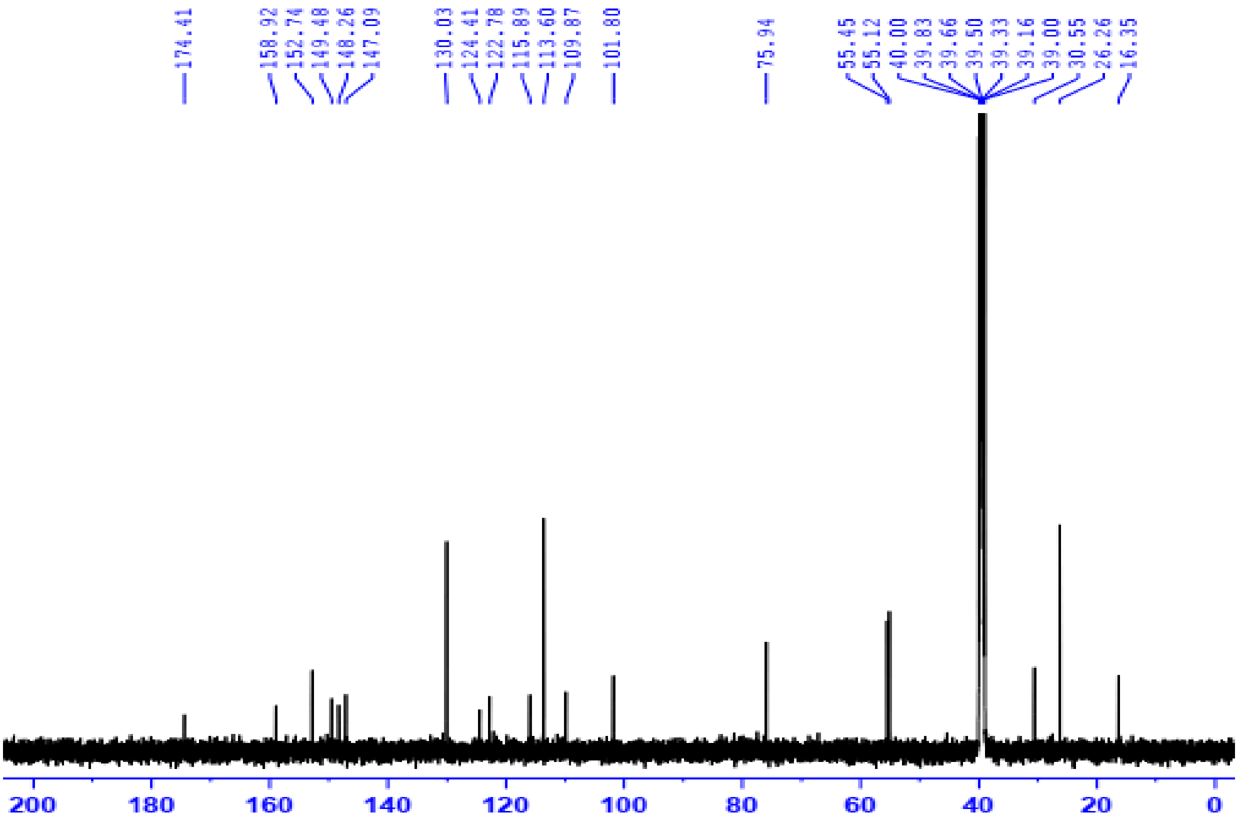

Figure S2. The ^{13}C NMR spectrum of **1** in DMSO-d_6

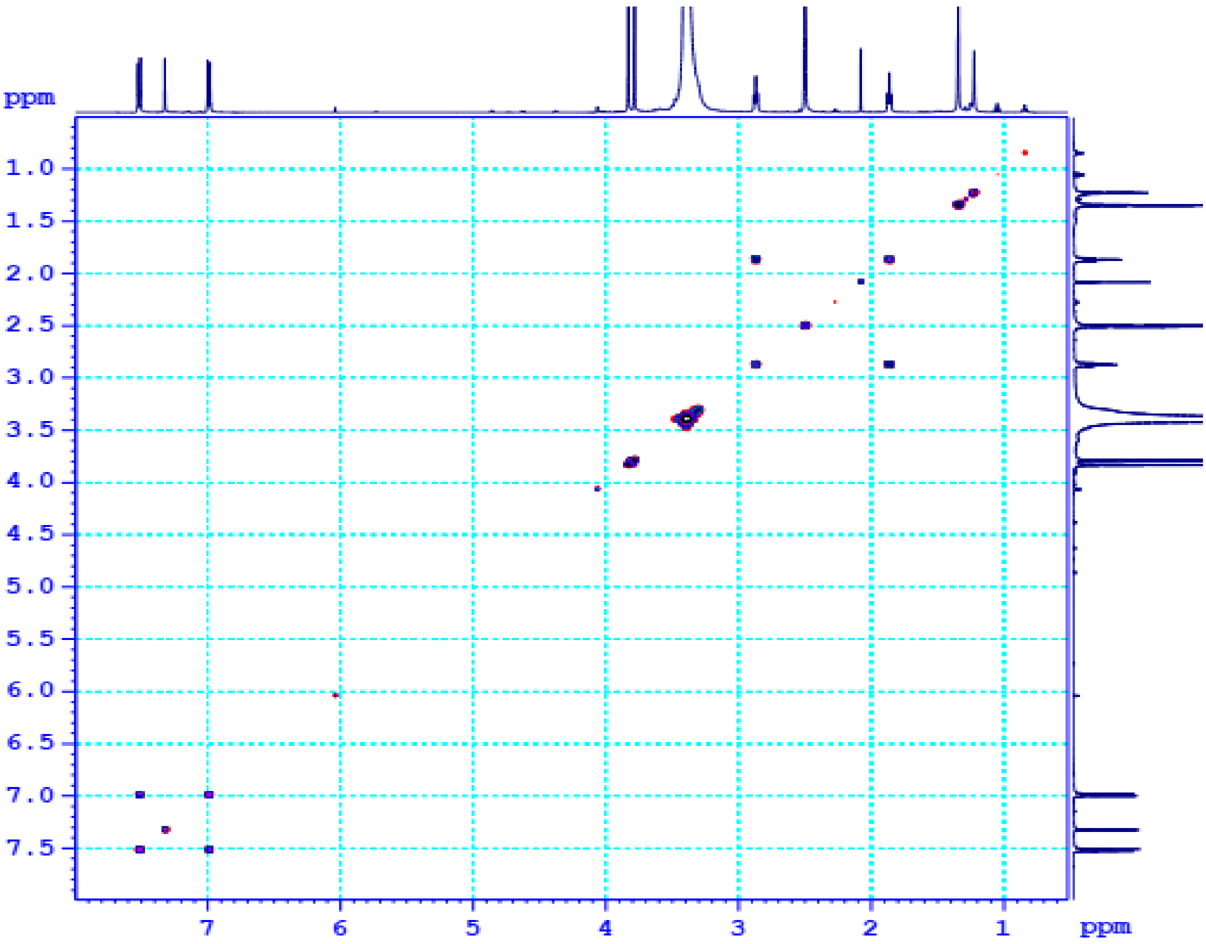

Figure S3. The COSY spectrum of **1** in DMSO-d_6

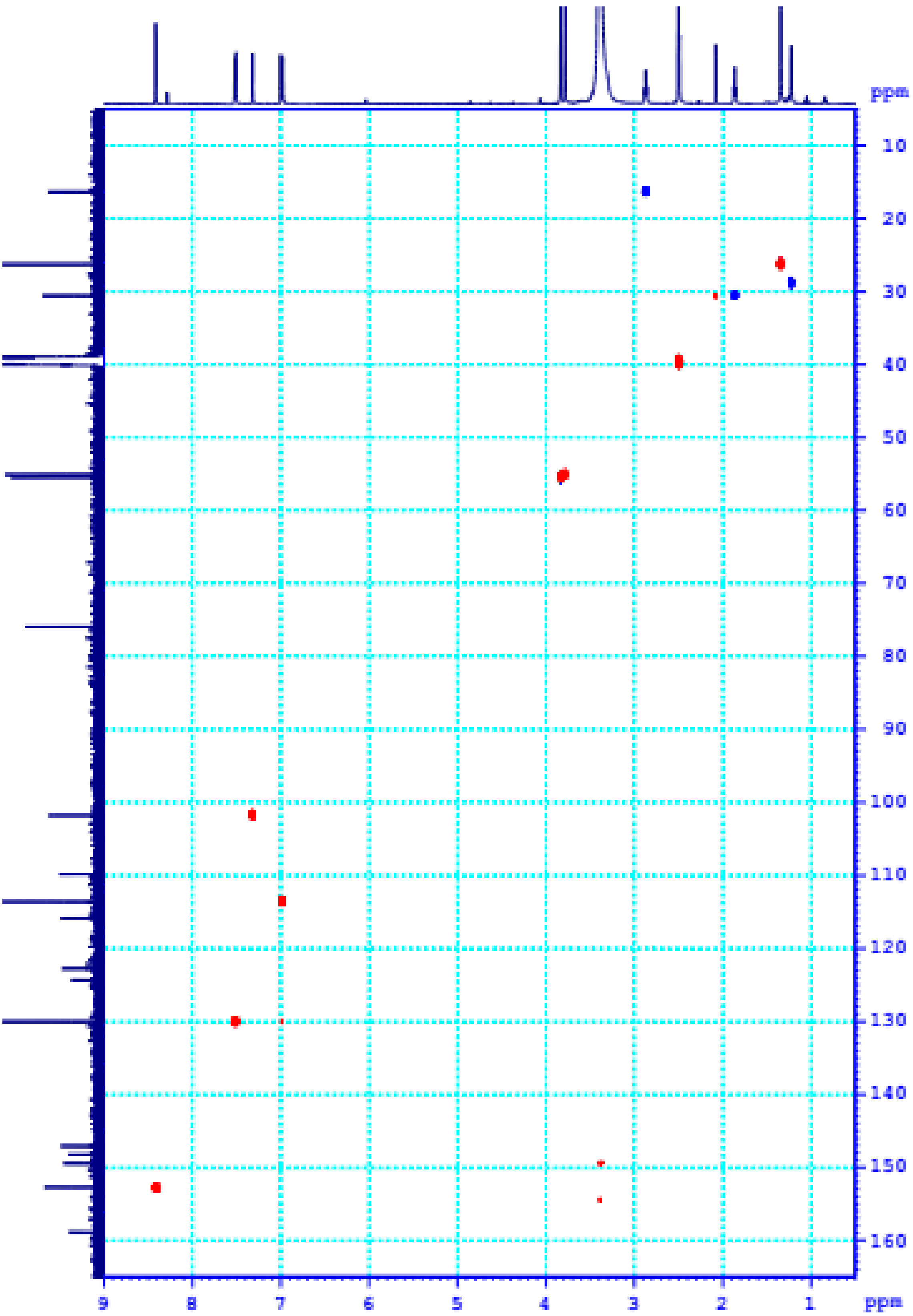

Figure S4. The HSQC spectrum of **1** in DMSO-d_6

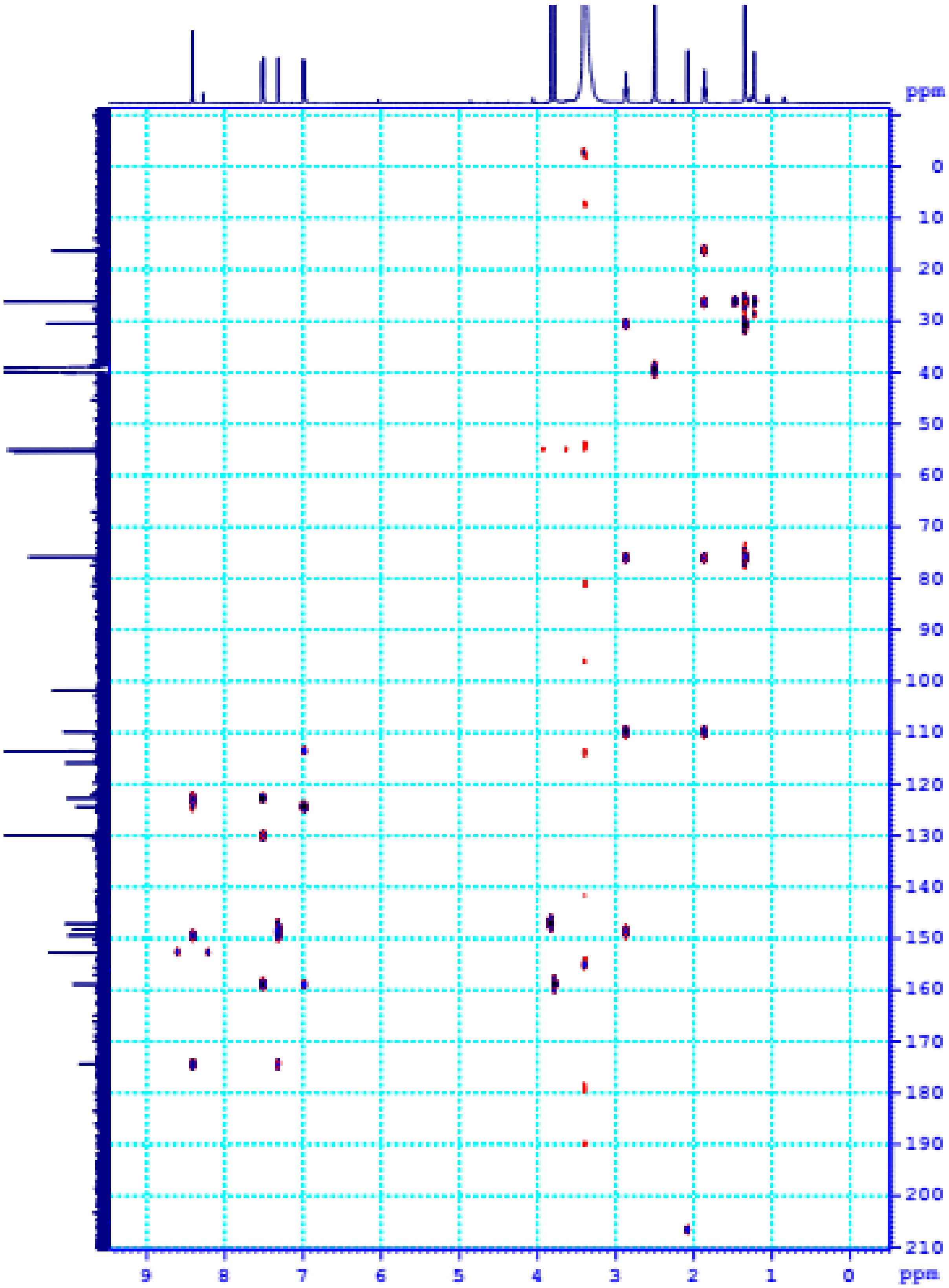

Figure S5. The HMBC spectrum of **1** in DMSO-d_6

ANALYSIS REPORT

Injection details

Sample name	LIEN204	*Vial position*	17
Sample file name	SER. wiff2 – LIEN	*Inject volume*	5.00
Acquisition date	01/09/2020 10:31:36 AM	*Acquisition method*	**ESI _POS_SCAN**
Operator	CB21261708	*Instrument name*	X500$_R$ QTOF

Full mass spectrum

Spectrum from LIEN204_(+)ESI.wiff2 (sample 1) - LIEN204_(+)ESI, +TOF MS (50 - 1500) from 0.153 min, noise filtered (noise multiplier = 1.5), Gaussian smoothed (0.5 points)

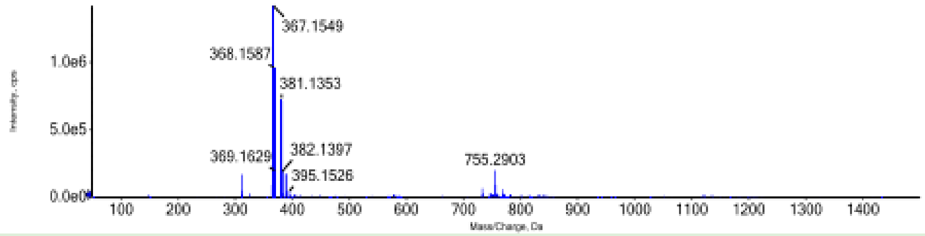

Expanded spectrum

Spectrum from LIEN204_(+)ESI.wiff2 (sample 1) - LIEN204_(+)ESI, +TOF MS (50 - 1500) from 0.153 min, noise filtered (noise multiplier = 1.5), Gaussian smoothed (0.5 points)

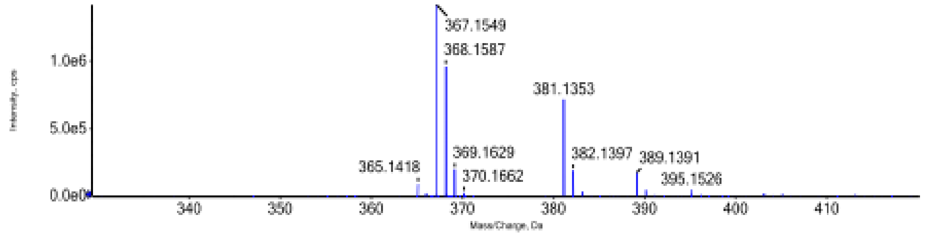

Figure S6. The HRESIMS spectrum of **1**

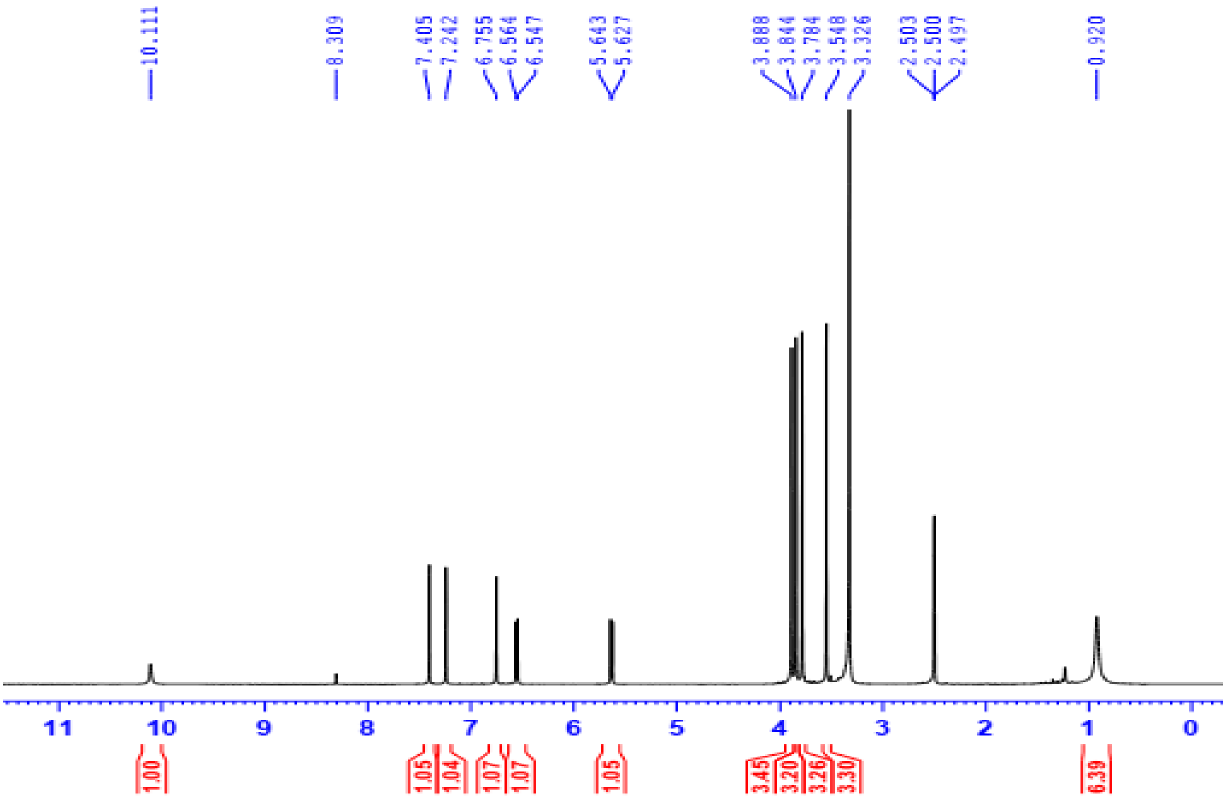

Figure S7. The ^{1}H NMR spectrum of **2** in DMSO-*d*6

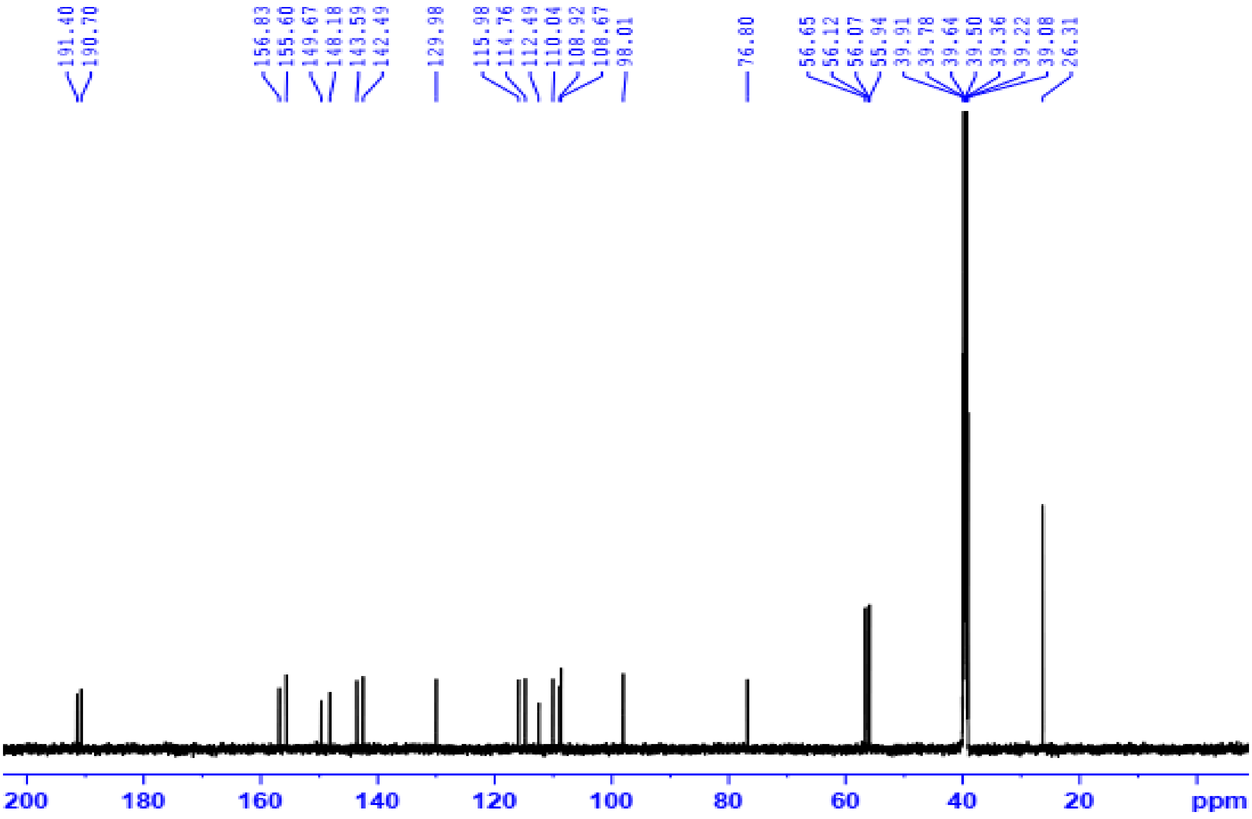

Figure S8. The ^{13}C NMR spectrum of **2** in DMSO-*d*6

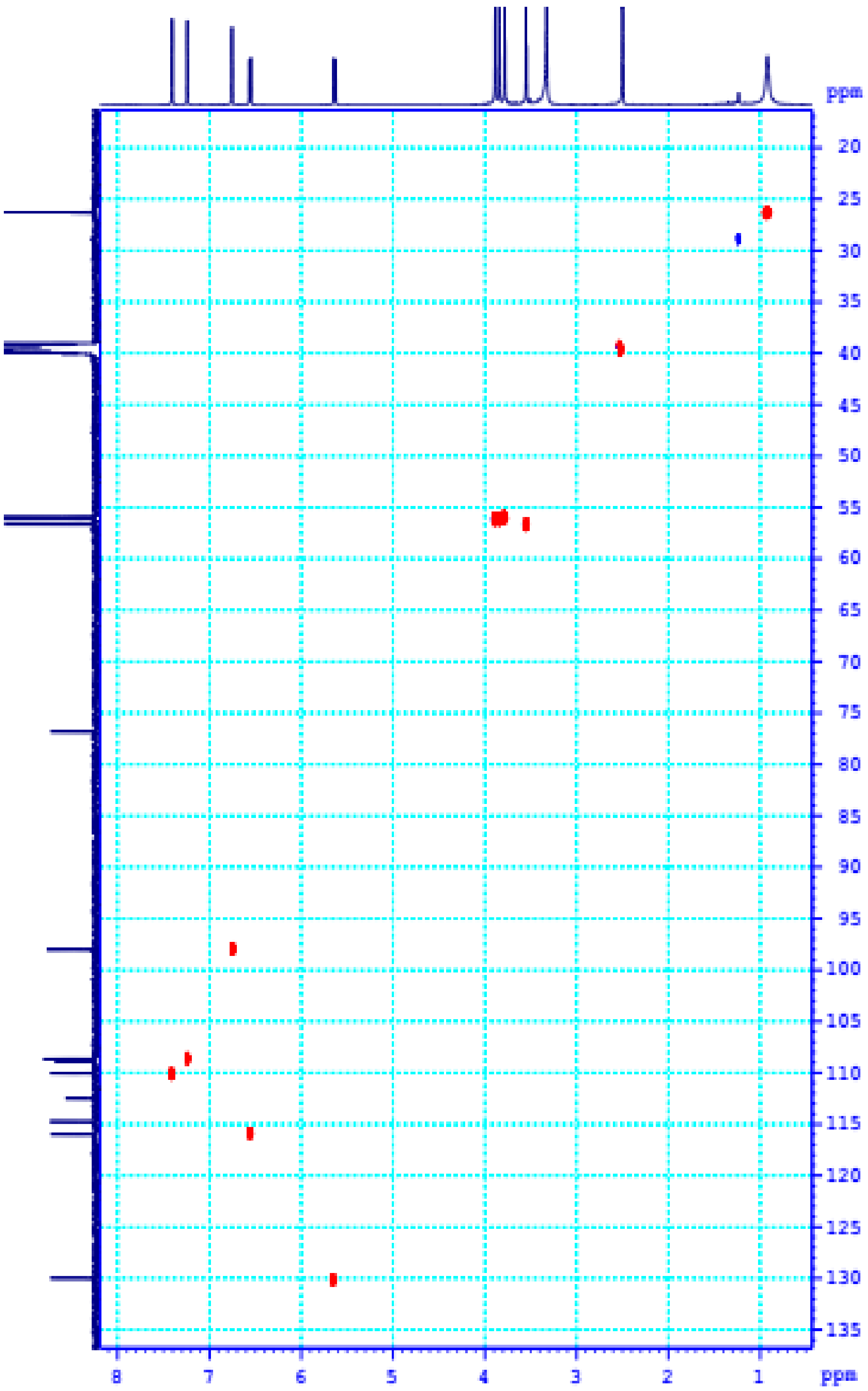

Figure S9. The HSQC spectrum of **2** in DMSO-d_6

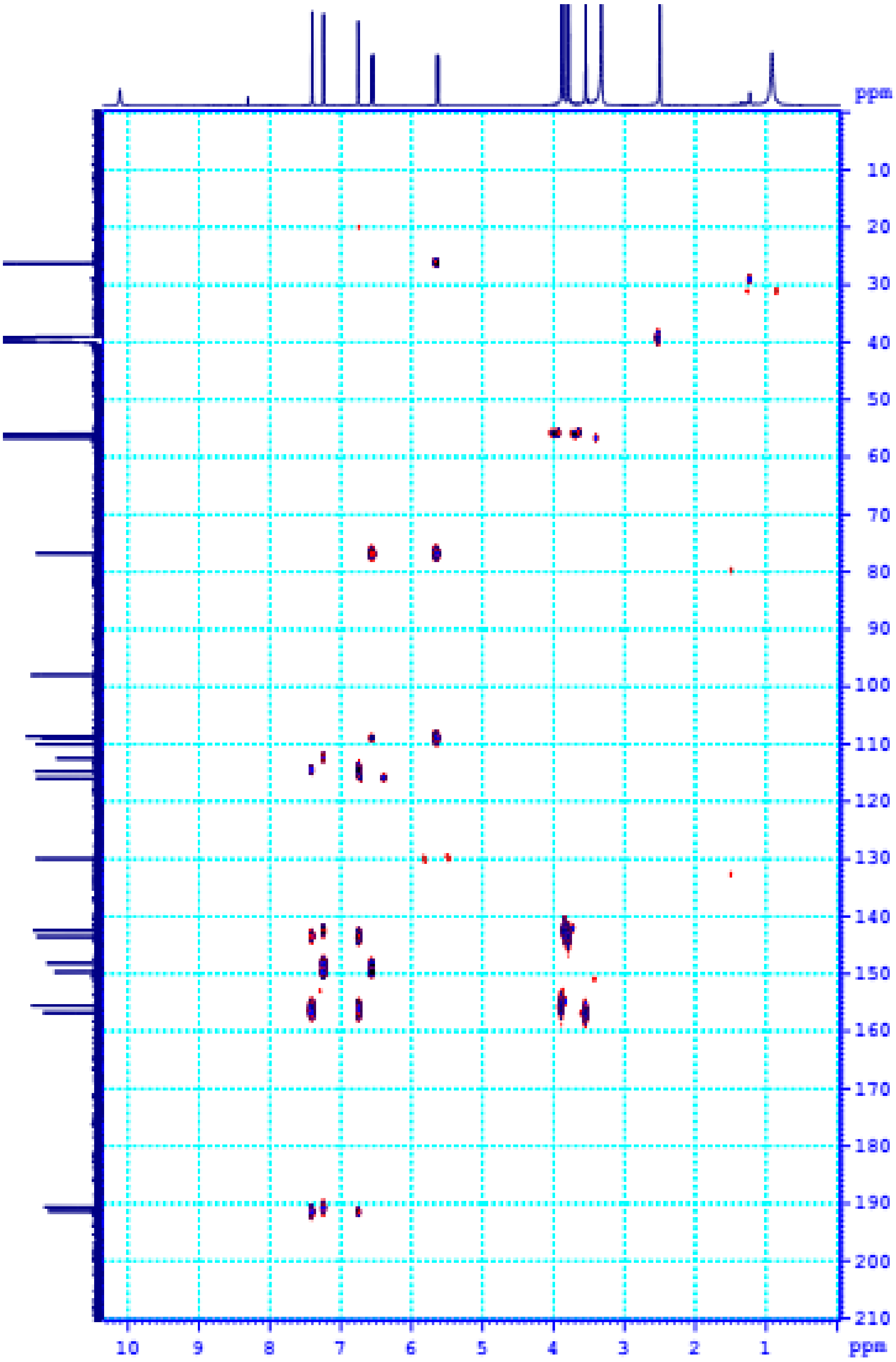

Figure S10. The HMBC spectrum of **2** in DMSO-d_6

ANALYSIS REPORT

Injection details

Sample name	LIEN 218	*Vial position*	12
Sample file name	SER. wiff2-LIEN	*Inject volume*	5.00
Acquisition date	26/05/2022 03:12:03 PM	*Acquisition method*	**ESI _POS_SCAN**
Operator	CB21261708	*Instrument name*	$X500_R$ QTOF

Full mass spectrum

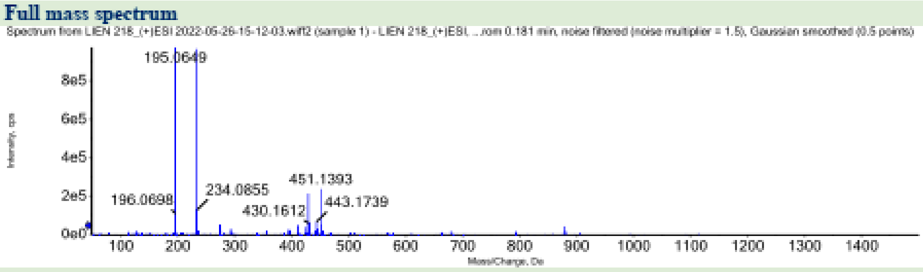

Expanded spectrum

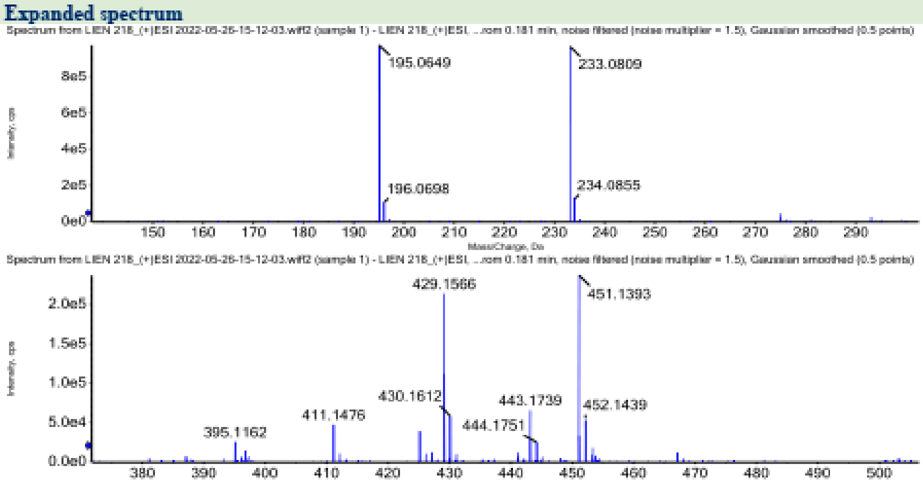

Figure S11. The HRESIMS spectrum of **2**

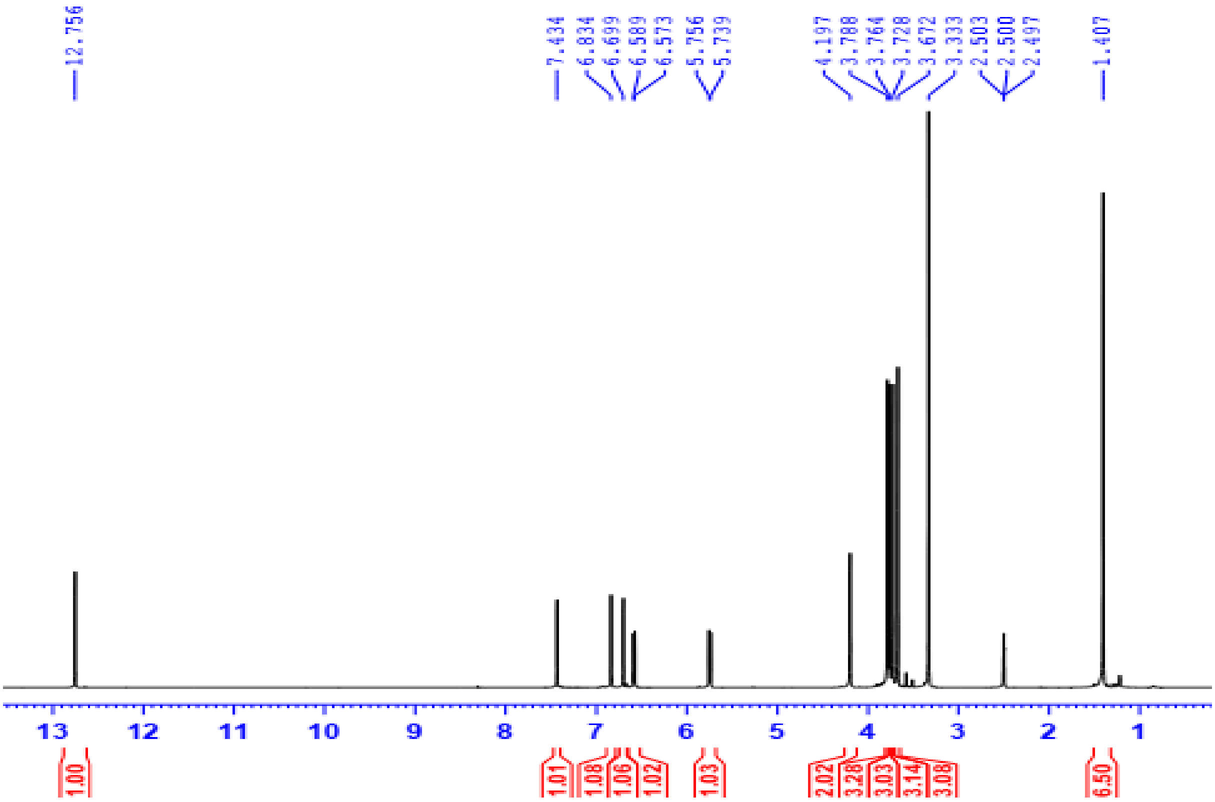

Figure S12. The ^{1}H NMR spectrum of **3** in DMSO-d_6

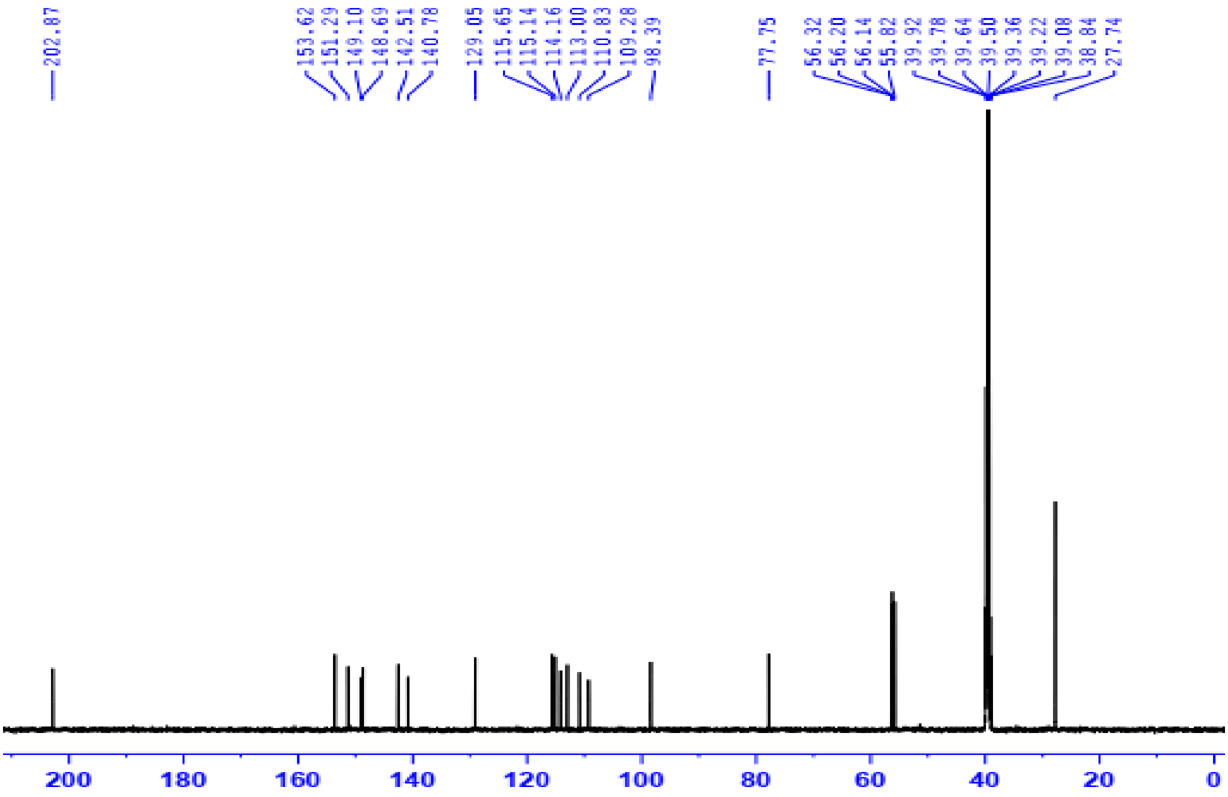

Figure S13. The ^{13}C NMR spectrum of **3** in DMSO-d_6

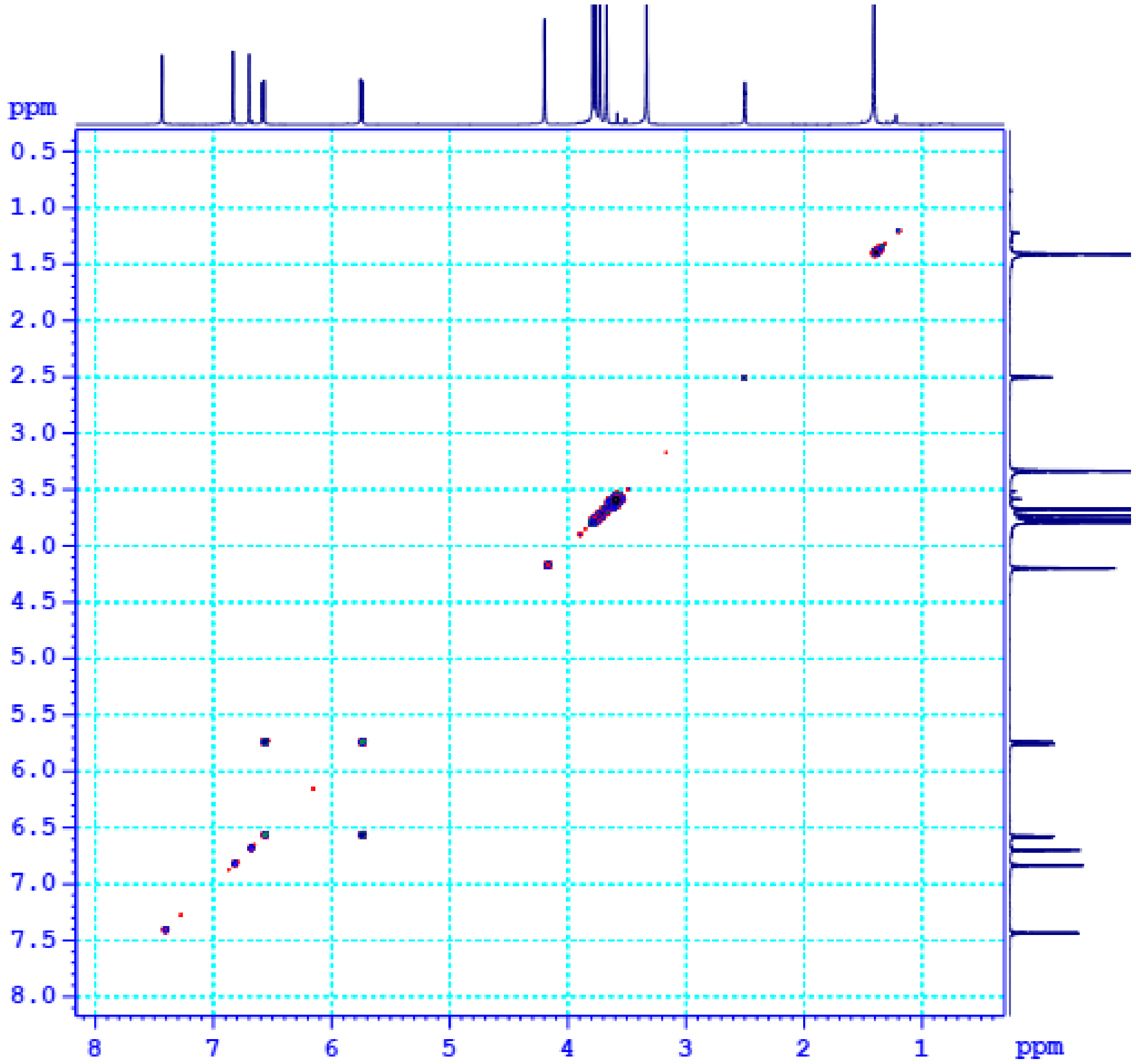

Figure S14. The COSY spectrum of **3** in DMSO-d_6

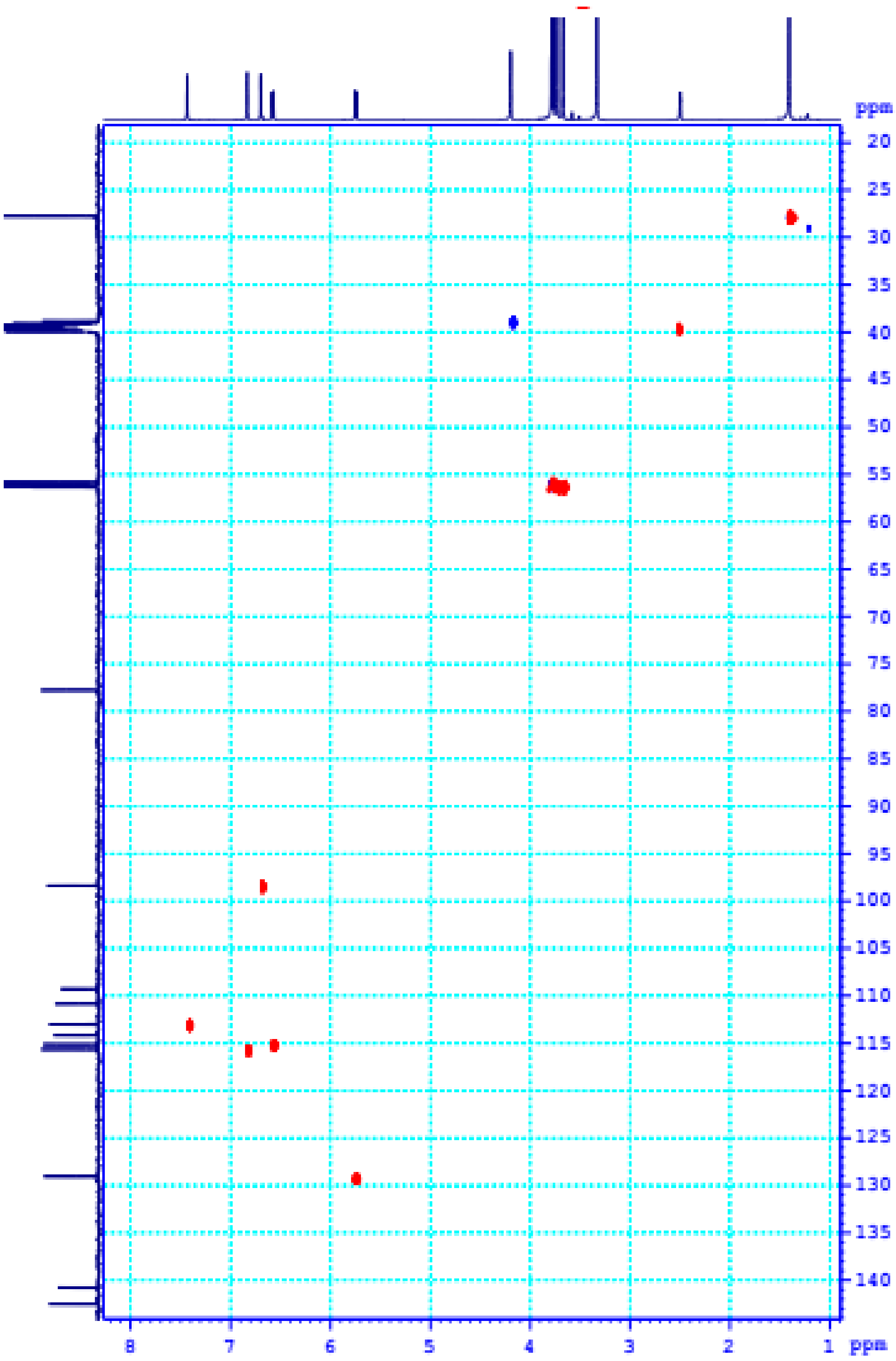

Figure S15. The HSQC spectrum of **3** in DMSO-d_6

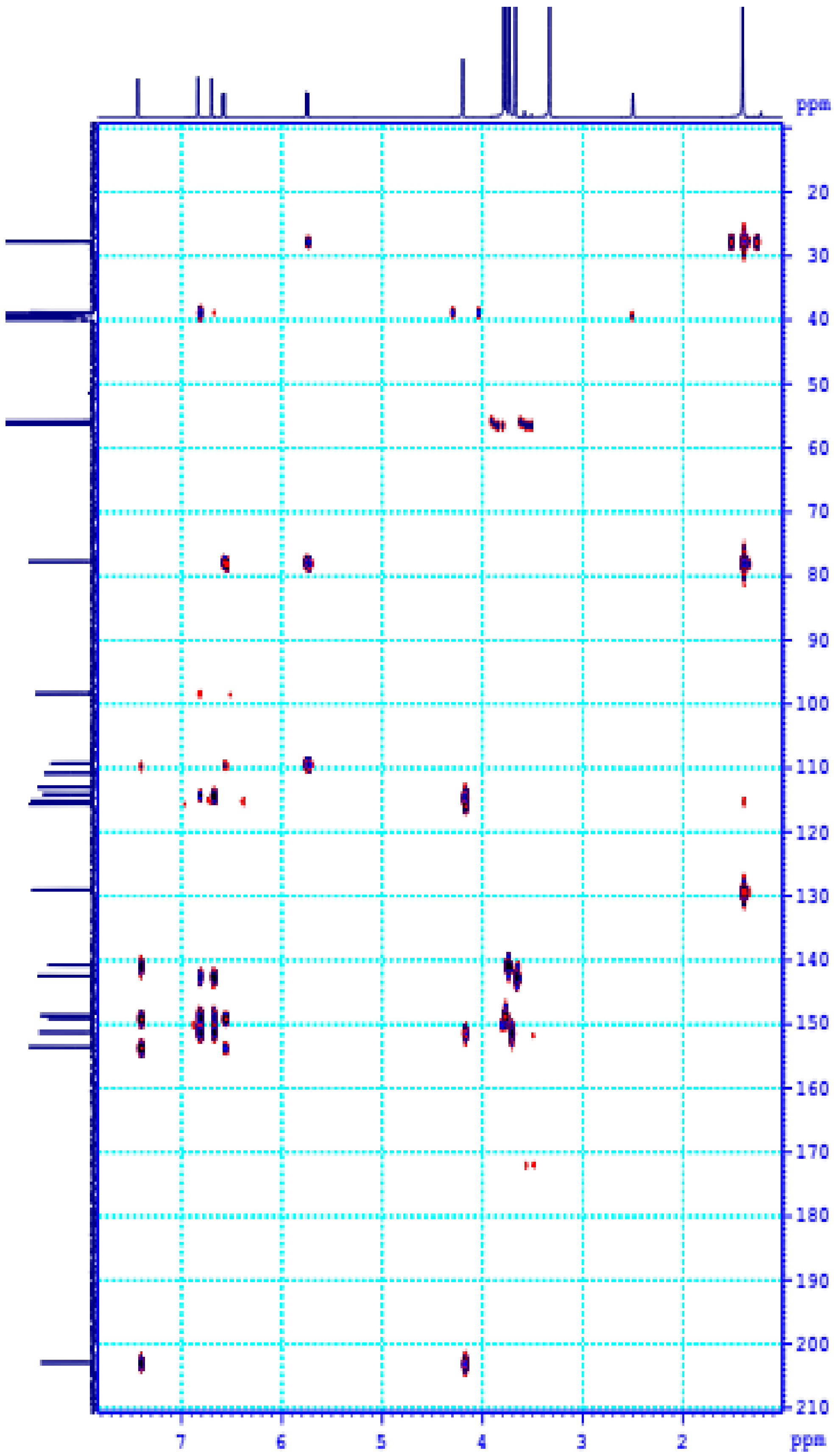

Figure S16. The HMBC spectrum of **3** in DMSO-d_6

Created with SCIEX OS 1.2

CENTER FOR RESEARCH AND TECHNOLOGY TRANSFER
CENTER FOR RESEARCH AND PHARMACEUTICAL TESTING
01, TL29 St., Dist 12, Ho Chi Minh City, Vietnam. Phone: (84) 907 070 939

ANALYSIS REPORT

Injection details

Sample name	LIEN 216	*Vial position*	11
Sample file name	SER. wiff2-LIEN	*Inject volume*	5.00
Acquisition date	26/05/2022 03:09:48 PM	*Acquisition method*	**ESI _POS_SCAN**
Operator	CB21261708	*Instrument name*	$X500_R$ QTOF

Full mass spectrum

Spectrum from LIEN 216_(+)ESI 2022-05-26-15-09-48.wiff2 (sample 1) - LIEN 216_(+)ESI, ...rom 0.171 min, noise filtered (noise multiplier = 1.5), Gaussian smoothed (0.5 points)

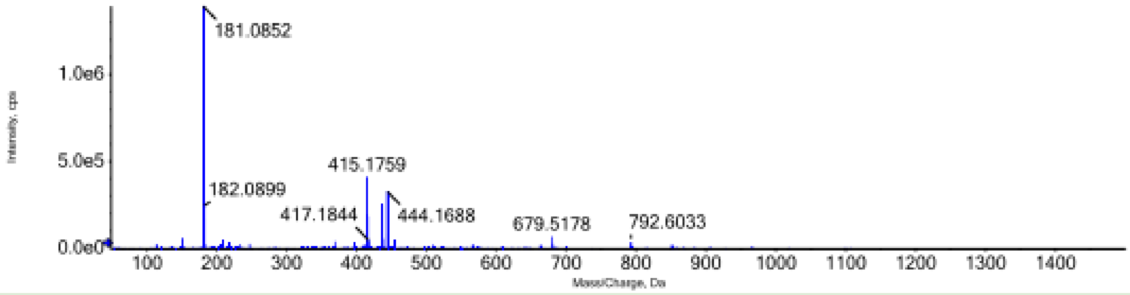

Expanded spectrum

Spectrum from LIEN 216_(+)ESI 2022-05-26-15-09-48.wiff2 (sample 1) - LIEN 216_(+)ESI, ...rom 0.171 min, noise filtered (noise multiplier = 1.5), Gaussian smoothed (0.5 points)

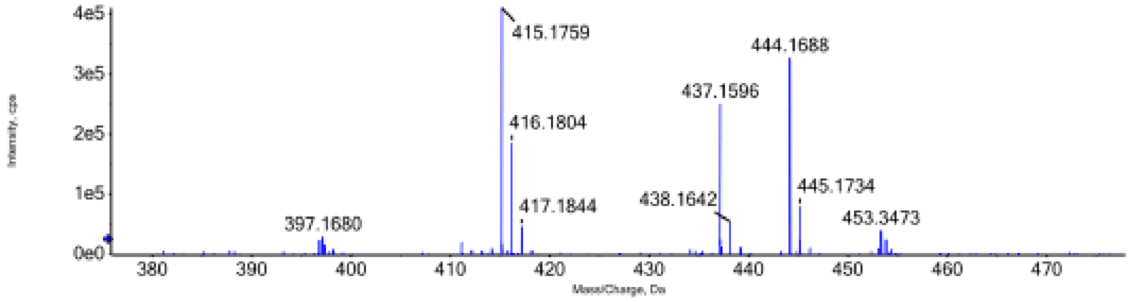

Figure S17. The HRESIMS spectrum of **3**

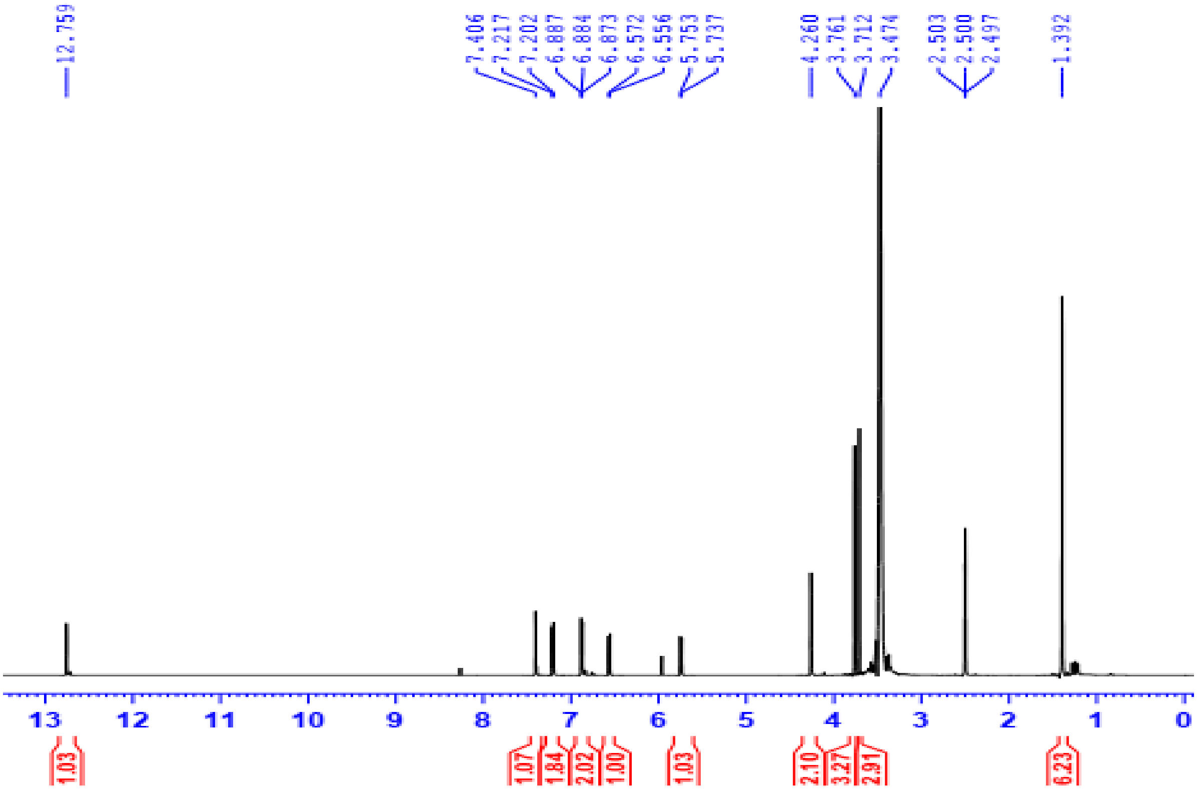

Figure S18. The ^{1}H NMR spectrum of **4** in DMSO-d_6

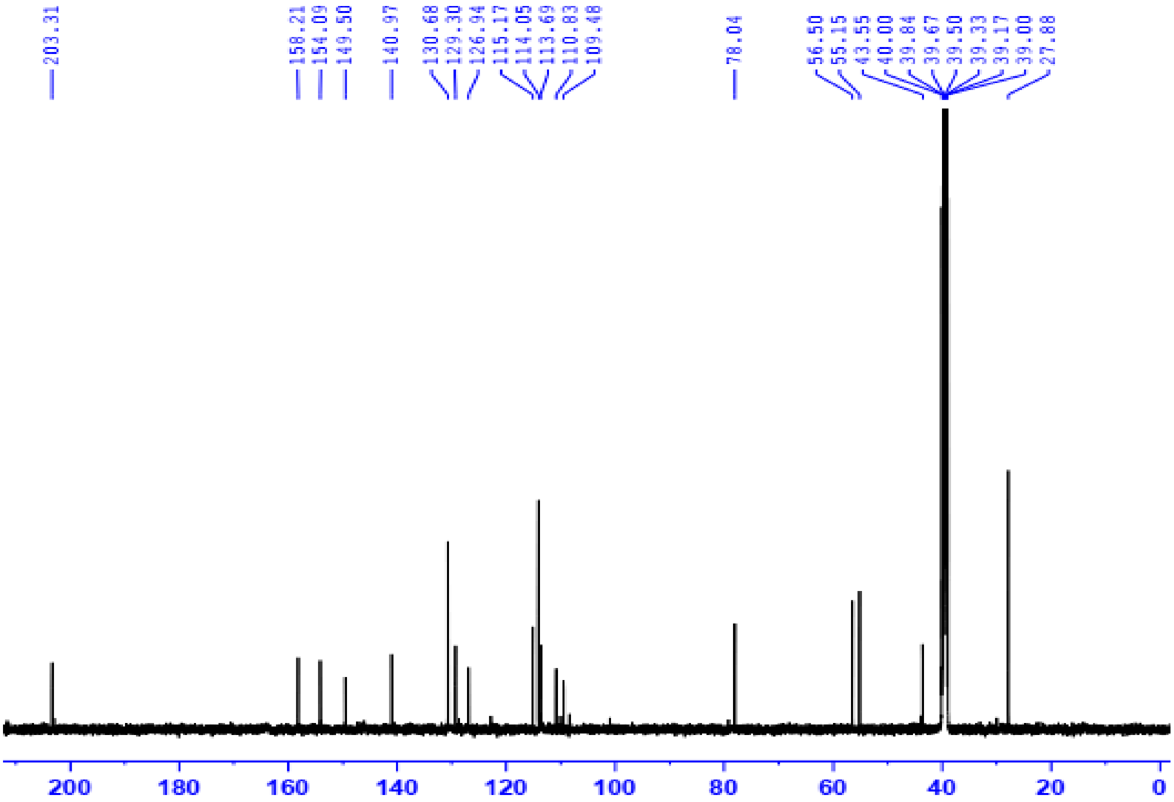

Figure S19. The ^{13}C NMR spectrum of **4** in DMSO-d_6

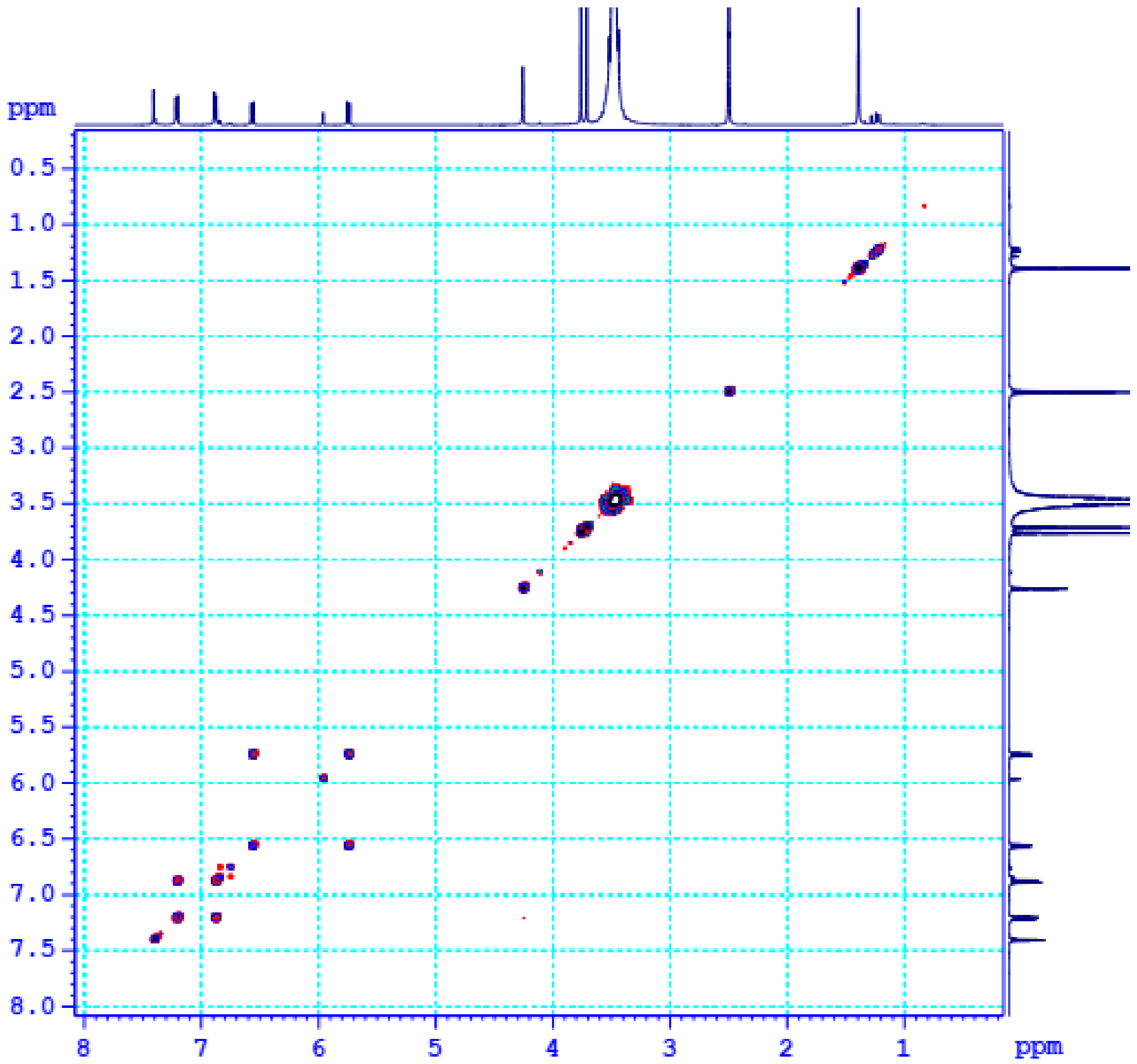

Figure S20. The COSY spectrum of **4** in DMSO-d_6

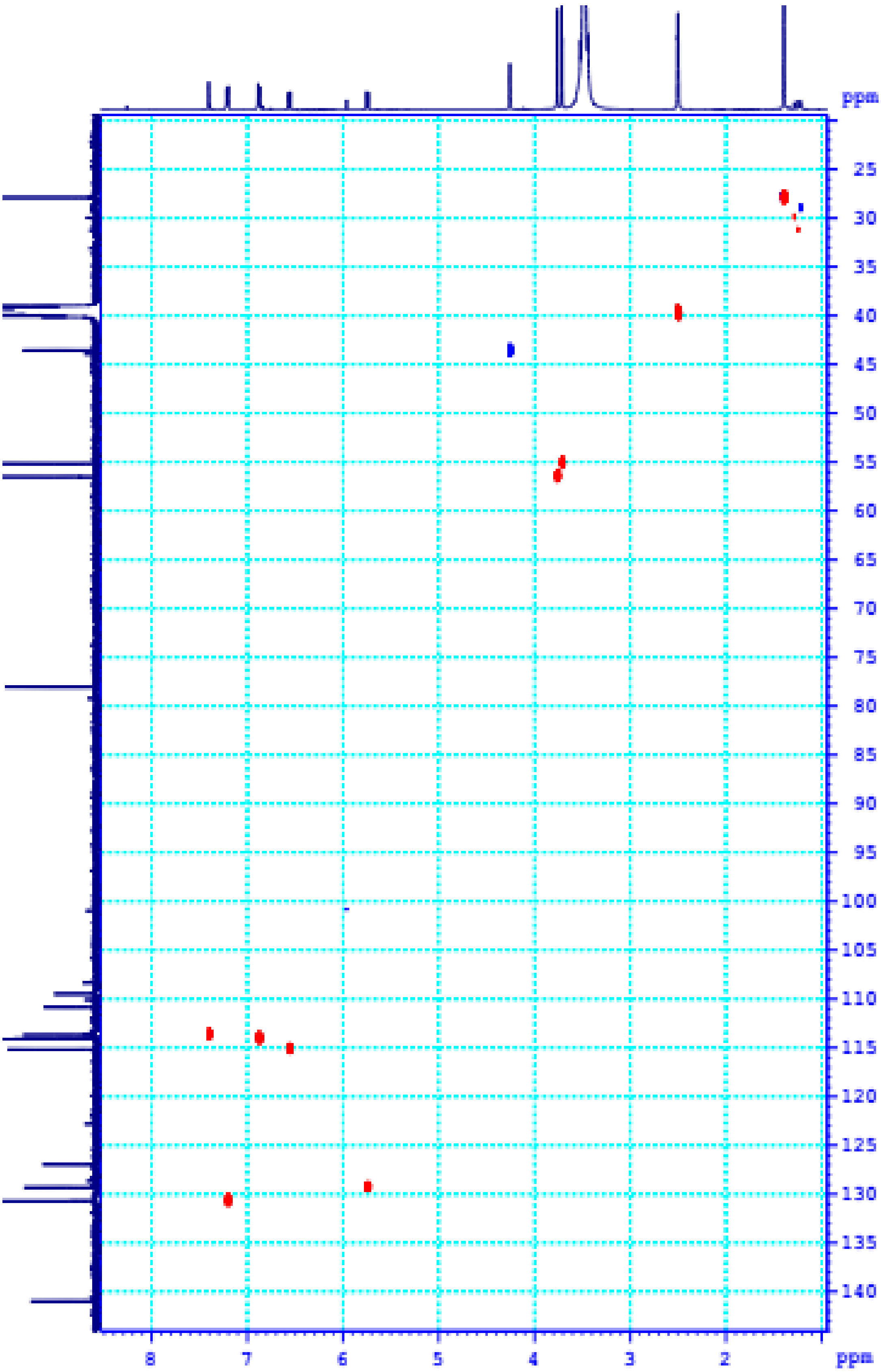

Figure S21. The HSQC spectrum of **4** in DMSO-d_6

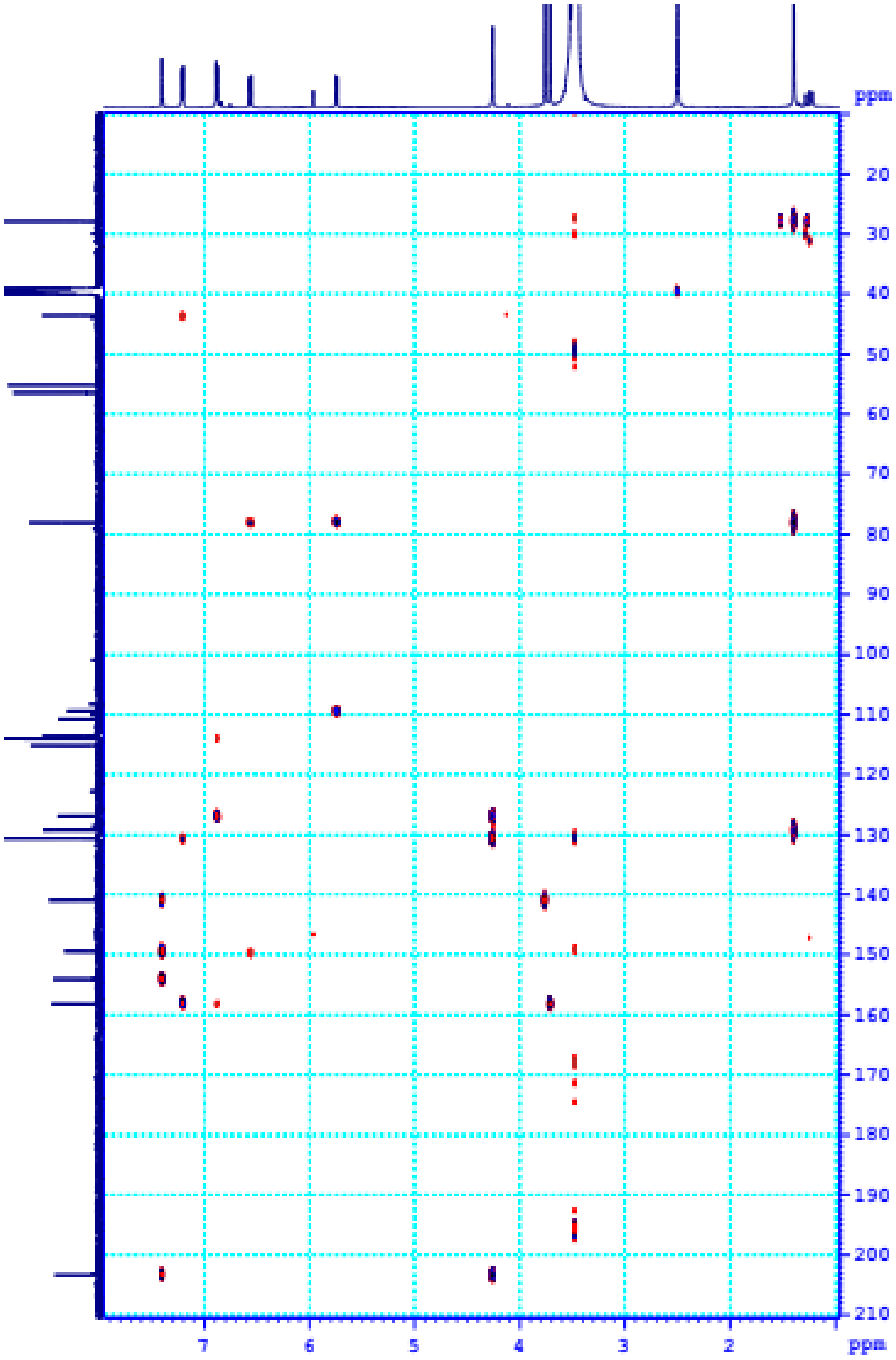

Figure S22. The HMBC spectrum of **4** in DMSO-d_6

ANALYSIS REPORT

Injection details

Sample name	LIEN_238	*Vial position*	23
Sample file name	SER. wiff2-LIEN	*Inject volume*	5.00
Acquisition date	18/01/2022 05:01:05 PM	*Acquisition method*	**ESI _NEG_SCAN**
Operator	CB21261708	*Instrument name*	X500R QTOF

Full mass spectrum

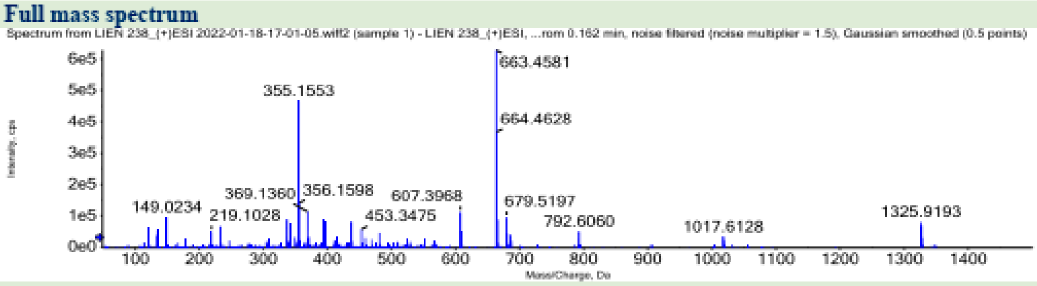

Expanded spectrum

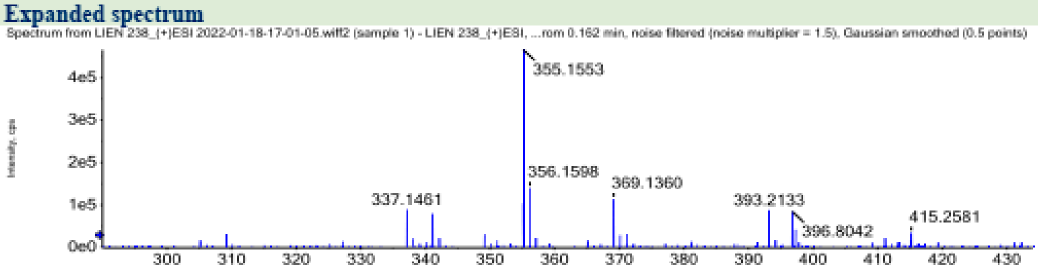

Figure S23. The HRESIMS spectrum of **4**
